# Numerical Computation for Gyrotactic Microorganisms in MHD Radiative Eyring–Powell Nanomaterial Flow by a Static/Moving Wedge with Darcy–Forchheimer Relation

**DOI:** 10.3390/mi13101768

**Published:** 2022-10-18

**Authors:** Muhammad Faizan Ahmed, A. Zaib, Farhan Ali, Omar T. Bafakeeh, El Sayed Mohamed Tag-ElDin, Kamel Guedri, Samia Elattar, Muhammad Ijaz Khan

**Affiliations:** 1 Department of Mathematical Sciences, Federal Urdu University of Arts, Science & Technology, Gulshan-e-Iqbal, Karachi 75300, Pakistan; 2 Department of Industrial Engineering, Jazan University, Jazan 82822, Saudi Arabia; 3 Faculty of Engineering and Technology, Future University in Egypt, New Cairo 11835, Egypt; 4 Mechanical Engineering Department, College of Engineering and Islamic Architecture, Umm Al-Qura University, P.O. Box 5555, Makkah 21955, Saudi Arabia; 5 Department of Industrial & Systems Engineering, College of Engineering, Princess Nourah Bint Abdulrahman University, P.O. Box 84428, Riyadh 11671, Saudi Arabia; 6 Department of Mathematics and Statistics, Riphah International University I-14, Islamabad 44000, Pakistan; 7 Department of Mechanical Engineering, Lebanese American University, Beirut 1102 2801, Lebanon

**Keywords:** Eyring–Powell nanofluid, magnetic field, nonlinear thermal radiation, motile microorganisms, static/moving wedge, Darcy–Forchheimer

## Abstract

The intention of this study is to carry out a numerical investigation of time-dependent magneto-hydro-dynamics (MHD) Eyring–Powell liquid by taking a moving/static wedge with Darcy-Forchheimer relation. Thermal radiation was taken into account for upcoming solar radiation, and the idea of bioconvection is also considered for regulating the unsystematic exertion of floating nanoparticles. The novel idea of this work was to stabilized nanoparticles through the bioconvection phenomena. Brownian motion and thermophoresis effects are combined in the most current revision of the nanofluid model. Fluid viscosity and thermal conductivity that depend on temperature are predominant. The extremely nonlinear system of equations comprising partial differential equations (PDEs) with the boundary conditions are converted into ordinary differential equations (ODEs) through an appropriate suitable approach. The reformed equations are then operated numerically with the use of the well-known Lobatto IIIa formula. The variations of different variables on velocity, concentration, temperature and motile microorganism graphs are discussed as well as force friction, the Nusselt, Sherwood, and the motile density organism numbers. It is observed that Forchheimer number Fr decline the velocity field in the case of static and moving wedge. Furthermore, the motile density profiles are deprecated by higher values of the bio convective Lewis number and Peclet number. Current results have been related to the literature indicated aforementioned and are found to be great achievement.

## 1. Introduction

The study of nanofluids has recently attracted the consideration of several researchers. The transmission of thermal energy can be sped up by employing nanofluids. Nanofluid is a mixture of tiny nanoparticles in a based fluid. In a comparison of base fluid, the thermal conductivity of nanofluid is significantly larger. Currently, nanofluids are having a significant impact on heat transmission. The heat of the exchange, a coolant of a nuclear reactor, microchip, drug delivery and many more. The term “nanofluid” was proposed by Choi [1]. The flow of nanofluid for thermophoresis and Brownian motion were then explored by Buongiorno [2] to identify the most suitable properties for enhancing heat transmission. The impact of radiation on Williamson nanoliquid moving toward a permeable media was utilized by Bhatti et al. [3]. Gireesha and colleagues [4] scrutinized the Eyring–Powell nanoliquid over a rotating moving surface of disk. In convective conditions, The stagnation point flow of Eyring–Powell nanofluid was discussed by Ali and Zaib [5]. The 3-D MHD Maxwell nanofluid with thermal behaviour was addressed by Vaidya et al. [6]. The thermal properties of nanofluid in the ferrofluid are discussed by Kumar et al. [7]. Chamkha et al. [8] made one more important addition concerning the joint impact of ion slip and Hall on MHD nano-liquid with a revolving disk through a permeable surface. With only a few numerical solutions, Sardar et al. [9] integrated the dual solution of Carreau nanofluid. The Williamson nanoliquid was numerically recruited toward a stretched plane by Danish et al. [10]. Ramesh et al. [11] explain the MHD Prandtl nano-liquid flow through the cavity. Alwatban et al. [12] presented a second-order slip condition with the effect of Eyring–Powell nanofluid using numerical simulation. Mekheimer and Ramadan [13] illustrated the existence of gyrotactic microorganisms on a stretching/shrinking plane to show the flow of Prandtl nano-liquid. The production and application of nanoliquid with base fluid were covered by Hussien et al. [14]. The second law with nanofluid compared with regular fluid was investigated by Hussain et al. [15]. Heat relocation on a hybrid nanofluid with a homogeneous/heterogeneous response was studied by Al-Kouzand co-workers. [16]. Al-Kouz et al. [17] and Mahanthesh et al. [18] studied dusty hybrid nanofluid through a vertical surface. The cavity-based 2D (two-dimensional) flow of nanoliquid with fin effects was planned by Al-Farhany et al. [19,20]. The Eyring–Powell nanoliquid exploitation using SWNTover an inclined plate was developed by Jamshed et al. [21].

Recently, the flow across a wedge region has received a great idea of attention owing to its manufacturing sectors and engineering fields in which it is used. Falkner and Skan [22] thought about this supposition in 1931 and established the Falkner–Skan mathematical formula. Rajagopal et al. [23] also studied the perturbation approach to examine the second-grade fluid of Falkner–Skan. Boundary layer flow of force convection using of heat transmission through a wedge was documented by Lin and Lin [24]. Kuo et al. [25] transmuted the differential expression over a wedge on the Falkner–Skan. Mishra et al. [26] examined the MHD flow of fluid with nanofluid passing a wedge. The micropolar fluid flow through a stretched and shifting wedge was thought out by Ishak et al. [27]. Ganganapalli et al. [28] examined the non-Newtonian (Casson) flow based on time toward a wedge per slip impact. Such liquid that passed a moving wedge feature of bioconvection was revealed by Raju et al. [29]. Tangent hyperbolic nanofluid with time dependent flow past a wedge plane was recently evaluated by Atif et al. [30]. Khan et al. [31] evaluated a dynamic wall-induced pressure gradient in the Falkner–Skan flow.

Bioconvection is extensively used in biotechnology, biofuels, and environmental systems. Microorganisms increase a fluid’s initial density and create a density gradient while swimming, which causes bioconvection. Numerous domains, including microcontrollers, bioinformatics, nanomaterials, and microfluidics, use bioconvection in various ways. The combination of nanotechnology and motile microorganisms, which improves the stability, heat transfer, and mass movement of nanomaterials, is another crucial aspect of bioconvection. Additionally, it has been applied biomedically to treat cancer. Newly, it has penetrated the gas industry. In their research on nanoliquid with bioconvection flow through a horizontal channels, Xu and Pop [32] provided numerical descriptions. The bioconvection flow of a water-based nano-liquid consisting gyrotactic microorganisms is originate via Siddiqa et al. [33]. Zuhra et al. [34] detected the non-Newtonian flowing over a vertical sheet subject to gyrotactic microorganisms. The unsteady flow of an Eyring–Powell nanomaterial containing gyrotactic microorganisms was planned by Mahdy et al. [35]. The thermal properties and viscosity with the bioconvection flow of nanofluid were further discussed by Xun et al. [36]. The impact of gyrotactic microorganisms on stratified nano-liquid was found by Alsaedi et al. [37].

Newtonian and non-Newtonian fluids (NNF) are identified by Newton’s law of viscosity. Shear stress and shear strain do not obey a linear relationship because these non-Newtonian materials are ample more complex than Newtonian liquids. Some examples of commonplace solutions and polymers that are non-Newtonian include mud, toothpaste, ketchup, blood, starch suspensions, and paints. Additionally, (NNF) [38,39,40] have gained special attention in the current era. Many rheological properties are connected to the significance of non-Newtonian fluids in numerous biological, engineering, and physical processes. It is noteworthy that several characteristics of the non-Newtonian fluid are not well described by a distinct relation. Therefore, several non-Newtonian models relating to its rheology have been suggested by investigators. One of these non-Newtonian fluids was the Eyring–Powell fluid model, which Eyring and Powell [41] introduced in 1944. Eyring–Powell fluid is the preferred non-Newtonian fluid since it’s based on a kinetic molecular model of liquids, not an empirical relationship. The vital importance of industrial fluids compels investigators to investigate their utilisation and heat flow obstacles. The Eyring–Powell nano liquid under homogeneous and heterogeneous past rotating disk was elaborated by Gholinia et al. [42]. The MHD flow Eyring–Powell nanofluid over the oscillatory surface is described by Khan et al. [43]. The Eyring–Powell nano-liquid on an inclined plane was seen through the work of Salawu and Ogunseye [44]. Abegunrin et al. [45] have used the Eyring–Powell flow (EPF) near a catalytic process. Rahimi et al. [46] intended statistically a technique to compute the flow of (EP) toward the linear stretching plane. The influence of the 3-D flow of (EP) nanofluid with chemical and activation energy past a slendering stretchable sheet was discussed via Reddy et al. [47].

The majority of prior literature referred to ordinary fluid with Newtonian fluid. EPF are scientifically essential and very much explored, EP as an ordinary fluid having MHD nanoliquids well as thermal radiative flow with microorganisms towards static/moving wedge interest us. The main goal of this endeavour is to improve heat transmission. Furthermore, it can be inferred from the aforementioned literature that the current research is unique and that no other analyses of this kind have been discussed. In the present exploration, the numeric result of converted ODE’s is providing the bvp4c method, bvp4c has three influences: a functioning system of ODE’s, a function (BCs) for exploring the residual in the boundary conditions. The impression of physical fluctuation on the velocity, heat, mass, and motile density of microorganisms is made visually and extensively described.

## 2. Materials Formulation

**Figure 1:**
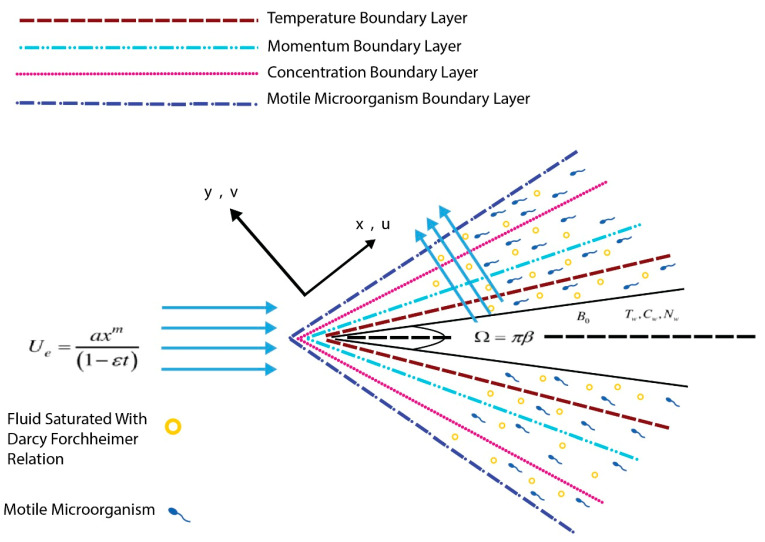
Schematic structure.

We assume that the (2D) flow of incompressible, laminar flow of bioconvection flow of Eyring–Powell nanoliquid through a wedge with Darcy–Forchheimer. Figure 1 below portrays the flow formation and system of coordinates. The moving-static considered in the the fluid flow. To maintain velocity $U_{w}=\frac{bx^{m}}{\left(1-\varepsilon t\right)}$ and upper surface velocity $U_{e}=\frac{ax^{m}}{\left(1-\varepsilon t\right)}$. Here $U_{w}\left(x,t\right)$ is greater than zero denotes a stretchable wedge and $\;U_{w}\left(x,t\right)$ ***is less than zero*** designates a dwindling wedge (see Figure 1). Note that a, b, m and ε  are coefficients with 0≤m≤1. Assumed that wedge angle =βπ. Furthermore, magnetic field strength $B=\frac{B_{0}x^{m-1/2}}{\sqrt{1-\varepsilon t}}$ is functional to the normal stretched plane. $T_{w},\;C_{w}$ and $N_{w}$ are the stable temperature, concentration and motile density at the plane, respectively. The temperature of ambient ($T_{\infty }),$ concontration ($C_{\infty })$ and motile density ($N_{\infty })$ are acquired as y→∞. The mathematical statement of concentration, energy, and momentum are expressed via the nanofluid Buongiorno model. The Buongiorno nanofluid model is essentially two-phased as opposed to the single-phase model of nanofluid, which means that the nanoparticles scattered into the base fluid vary from the wall to the outer boundary and must be solved concurrently with the base fluid. The use of the Buongiorno nanofluid model allows for the analysis of the well-known characteristics of Brownian motion and thermophoresis. The following assumptions are also made. In a wedge geometry, Eyring–Powell nanofluid flow is laminar, unsteady, and incompressibly. Chemical reaction $\;\left(Cr\right)$ and thermal radiation $\left(Rd\right)$ are both included in the formulation.

The stress tensor for Eyring–Powell fluid model is defined by the form:

(1) Λ=−PI+τ

Note that *P* and Λ symbolize as basic stress tensors and extra stress tensors and Λ is defined as [20]:

(2) $$\tau_{ij}=\mu \frac{\partial u_{i}}{\partial x_{j}}+\frac{1}{\Lambda }\sinh^{-1}\left(\frac{1}{d}\frac{\partial u_{i}}{\partial x_{j}}\right)$$

(3) $$\sinh^{-1}\left(\frac{1}{d}\frac{\partial u_{i}}{\partial x_{j}}\right)≅\frac{1}{d}\frac{\partial u_{i}}{\partial x_{j}}-\frac{1}{6}{\left(\frac{1}{d}\frac{\partial u_{i}}{\partial x_{j}}\right)}^{3},\left|\frac{1}{d}\frac{\partial u_{i}}{\partial x_{j}}\right|<<1,$$

Equation (2) describes the form

(4) $$\tau_{ij}=\left(\mu +\frac{1}{\Lambda d}\right)\frac{\partial u_{i}}{\partial x_{j}}-\frac{1}{6\Lambda d^{3}}{\left(\frac{\partial u_{i}}{\partial x_{j}}\right)}^{3}$$

Taking into account the aforementioned hypotheses, the mathematical form is [20,36,37]

(5) $$\frac{\partial u}{\partial x}+\frac{\partial v}{\partial y}=0,$$

(6) $$\frac{\partial u}{\partial t}+u\frac{\partial u}{\partial x}+v\frac{\partial u}{\partial y}=\frac{\partial Ue}{\partial t}+U_{e}\frac{\partial U_{e}}{\partial x}+\left(v+\frac{1}{\rho \Lambda d}\right)\frac{\partial^{2}u}{\partial y^{2}}-\frac{1}{2\rho \Lambda c^{3}}{\left(\frac{\partial u}{\partial y}\right)}^{2}\frac{\partial^{2}u}{\partial y^{2}}-\frac{\sigma B_{0}^{2}}{\rho }\left(u-U_{e}\right)-\frac{v}{K}\left(u-U_{e}\right)-Fr\left(u^{2}-U_{e}{}^{2}\right)=0,$$

(7) $$\frac{\partial T}{\partial t}+u\frac{\partial T}{\partial x}+v\frac{\partial T}{\partial y}=\alpha_{m}\frac{\partial^{2}T}{\partial y^{2}}+\tau \left(D_{B}\frac{\partial C}{\partial y}\frac{\partial T}{\partial y}+\frac{D_{T}}{T_{\infty }}{\left(\frac{\partial T}{\partial y}\right)}^{2}\right)-\frac{1}{{\left(\rho c\right)}_{f}}\frac{\partial q_{r}}{\partial y},$$

(8) $$\frac{\partial C}{\partial t}+u\frac{\partial C}{\partial x}+v\frac{\partial C}{\partial y}=D_{B}\frac{\partial^{2}C}{\partial y^{2}}+\frac{D_{T}}{T_{\infty }}\left(\frac{\partial^{2}T}{\partial y^{2}}\right)-\kappa_{0}\left(C_{w}-C_{\infty }\right),$$

(9) $$\frac{\partial \chi }{\partial t}+u\frac{\partial \chi }{\partial x}+v\frac{\partial \chi }{\partial y}+b\frac{W_{c}}{C_{0}}\frac{\partial }{\partial y}\left(\chi \frac{\partial C}{\partial y}\right)=D_{m}\frac{\partial^{2}\chi }{\partial y^{2}},$$

The boundary conditions are

(10) $$\left.\begin{array}{c} u=U_{w}=\lambda U_{e},\;v=v_{w},-k\frac{\partial T}{\partial ŷ}=h\left(t\right)\left(T_{f}-T\right),\;\\ {C=C}_{w}\left(x,t\right){,\;N=N}_{w}\left(x,t\right)\;\mathrm{at}\;y=0,\\ u\to U_{e},\;T\to T_{\infty },\;C\to C_{\infty },\;N\to N_{\infty }\mathrm{at}\;y\to \infty . \end{array}\right\}$$

where

(11) $$T_{w}\left(x,t\right)=T_{\infty }+\frac{T_{0}U_{w}x}{v{\left(1-\varepsilon t\right)}^{\frac{1}{2}}},C_{w}\left(x,t\right)=C_{\infty }+\frac{C_{0}U_{w}x}{v{\left(1-\varepsilon t\right)}^{\frac{1}{2}}},$$

(12) $$\left.\begin{array}{c} U_{w}=\frac{ax^{m}}{1-\varepsilon t}v_{w}\left(x,t\right)=-S\sqrt{\frac{\left(m+1\right)}{2}\frac{vue}{x}}\\ q_{r}=\frac{-4\sigma^{*}}{3k^{*}}\frac{\partial T^{4}}{\partial z}=-\frac{-16\sigma^{*}}{3k^{*}}T^{3}\frac{\partial T}{\partial z}, \end{array}\right\}$$

The subsequent transformation is

(13) $$\left.\begin{array}{c} u=U_{e}f^{\prime }\left(\eta \right),v=-\sqrt{\left(\frac{m+1}{2}\right)\frac{vU_{e}}{x}}\left[f\left(\eta \right)+\frac{m-1}{m+1}\eta f^{\prime }\left(\eta \right)\right]\\ \theta \left(\eta \right)=\frac{T-T_{\infty }}{T_{w}-T_{\infty }},\phi \left(\eta \right)=\frac{C-C_{\infty }}{C_{w}-C_{\infty }},\;\eta =y\sqrt{\frac{\left(m+1\right)U_{e}}{2vx}} \end{array}\right\}$$

Equation (13) is viewed from expressions (6)–(12) as:

(14) $$\begin{array}{c} \left(1+W\right)f^{\prime \prime \prime }+\beta \left(1-{f^{\prime }}^{2}\right)+ff^{\prime \prime }-W\gamma {f^{\prime \prime }}^{2}f^{\prime \prime \prime }-\epsilon \left(2-\beta \right)\left(\frac{\eta }{2}f^{\prime \prime }+f^{\prime }-1\right)-\\ M\left(2-\beta \right)\left(f^{\prime }-1\right)+\kappa \left(2-\beta \right)\left(f^{\prime }-1\right)+Fr\left(2-\beta \right)\left({f^{\prime }}^{2}-1\right)=0, \end{array}$$

(15) $$\theta^{\prime \prime }\left(1+\frac{4}{3}Rd\right)+2\mathrm{Pr}\left(\frac{f\theta^{\prime }}{2}-f^{\prime }\theta \right)+\mathrm{Pr}\left(\frac{2Nb\theta^{\prime }\phi^{\prime }+2Nt{\theta^{\prime }}^{2}-\left(2-\beta \right)\epsilon \left(\eta \theta^{\prime }+3\theta \right)}{2}\right)=0,$$

(16) $$\phi^{\prime \prime }+Sc\left(\left(f\phi^{\prime }-2f^{\prime }\phi \right)-\frac{\epsilon }{2}\left(2-\beta \right)\left(\eta \phi^{\prime }+3\phi \right)\right)+\theta^{\prime \prime }\frac{Nt}{Nb}-ScCh\phi =0,$$

(17) $$N^{\prime \prime }-Pe\phi^{\prime }N^{\prime }-Pe\phi^{\prime \prime }\left(N+\varpi \right)+2Lb\left(\frac{fN^{\prime }}{2}-f\prime N-\frac{\epsilon }{2}\left(2-\beta \right)(\eta N^{\prime }+3N\right)=0,$$

The transmuted boundary conditions are

(18) $$\left.\begin{array}{c} f\left(\eta \right)=S,f^{\prime }\left(\eta \right)=0,\theta^{\prime }\left(\eta \right)=-Bi\left(1-\theta \left(\eta \right)\right),\phi^{\prime }\left(\eta \right)=1,N\left(\eta \right)=1at\;\eta =0\\ f^{\prime }\left(\eta \right)=1,\theta \left(\eta \right)=0,\phi \left(\eta \right)=0,N\left(\eta \right)=0at\;\eta =\infty \end{array}\right\}\left(\mathrm{Static}\;\mathrm{wedge}\right)$$

(19) $$\left.\begin{array}{c} f\left(\eta \right)=S,f^{\prime }\left(\eta \right)=\lambda ,\theta^{\prime }\left(\eta \right)=-\gamma \left(1-\theta \left(\eta \right)\right),\phi^{\prime }\left(\eta \right)=1,N\left(\eta \right)=1at\eta =0\\ f^{\prime }\left(\eta \right)=1,\theta \left(\eta \right)=0,\phi \left(\eta \right)=0,N\left(\eta \right)=0.at\eta \to \infty \end{array}\right\}\left(\mathrm{Moving}\;\mathrm{wedge}\right)$$

The coefficients are

$$\left\{\begin{array}{c} \frac{a^{3}x^{2}}{\sqrt{\frac{\left(m+1\right)U_{e}}{2vx}}}=\gamma ,\;\frac{1}{\mu \Lambda d}=W,\;\frac{b}{ax^{m-1}}=\epsilon ,\mathrm{Pr}=\frac{v}{\alpha },\lambda =\frac{U_{w}}{U_{e}},\;Nb=\frac{\tau D_{B}\left(C_{w}-C_{\infty }\right)}{v},\\ Nt=\frac{\tau D_{T}\left(T_{w}-T_{\infty }\right)}{T_{\infty }v},\;Bi=\frac{h}{k\sqrt{\frac{\left(m+1\right)U_{e}}{2vx}}},\;M=\frac{\sigma B_{0}^{2}}{\rho a},\;\theta_{w}=\frac{T_{w}}{T_{\infty }},\;Rd=\frac{4\sigma^{*}T_{\infty }^{3}}{kk^{*}},\\ \;Pe=\frac{bW_{c}}{D_{m}},\varpi =\frac{\chi_{\infty }}{\chi_{m}-\chi_{\infty }},\;Sc=\frac{v}{D_{B}},Fr=\frac{c_{b}}{\sqrt{k_{2}}},\;\kappa =\frac{\upsilon l}{k_{2}u_{\circ }}. \end{array}\right\}$$

### Engineering Quantities

The $C_{fx}$, $Nu_{x}$,$\;Sh_{x}$ and Nn is designated as:

(20) $$C_{fx}=\frac{\tau_{w}}{\rho u_{w}^{2}},\;Nu_{x}=\frac{xq_{w}}{k\left(T_{w}-T_{\infty }\right)},\;Sh_{x}=\frac{xq_{m}}{D_{b}\left(C_{w}-C_{\infty }\right)},Nn=\frac{xq_{n}}{D_{m}\left(\chi_{w}-\chi_{\infty }\right)}$$

The $\tau_{w}$ characterize shear stress wall, $q_{w}$ indicate heat, $q_{m}$ denote mass, and $\;q_{n}$ signifies density of motile microorganisms are as follows

(21) $$\tau_{w}{\left.=\left(\mu +\frac{1}{\beta d}\right)\frac{\partial u}{\partial y}-\frac{1}{6}{\left(\frac{1}{d}\frac{\partial u}{\partial y}\right)}^{3}\right|}_{y=0},\;q_{w}=\left.-k\left(\frac{\partial T}{\partial y}\right)\right|,q_{m}={\left.-k\left(\frac{\partial C}{\partial y}\right)\right|}_{y=0},q_{n}={\left.-D_{m}\left(\frac{\partial \chi }{\partial y}\right)\right|}_{y=0}$$

Using Equation (13) and Equations (22)–(25) are

(22) (Rex)1/2Cf2−β=1+Wf″0−W3γf″30

(23) $$\frac{Nu_{x}}{Re_{x}^{1/2}}\sqrt{2-\beta }=-\theta^{\prime }\left(0\right)\left[1+\frac{4}{3}Rd\right],\;$$

(24) $$\frac{Sh_{x}}{Re_{x}^{1/2}}\sqrt{2-\beta }=-\phi^{\prime }\left(0\right),$$

(25) $$\frac{Nn}{Re_{x}^{1/2}}\sqrt{2-\beta }=-N^{\prime }\left(0\right).$$

where Rex=xUev denotes the Reynolds number.

## 3. Solution Strategy

The shooting method has been applied to solve nonlinear Equations (14)–(17) together with boundary conditions (18)–(19) to assign the following numerous unknowns:

(26) $$\begin{array}{c} \begin{array}{c} f=\Omega_{1},\;f^{\prime }=\Omega_{2},\;f^{\prime \prime }=\Omega_{3},\;f^{\prime \prime \prime }=\;{\Omega^{\prime }}_{3}\\ \theta =\Omega_{4},\;\theta^{\prime }=\Omega_{5},\;\theta^{\prime \prime }={\Omega^{\prime }}_{5} \end{array}\\ \phi =\Omega_{6},\phi^{\prime }=\Omega_{7},\phi^{\prime \prime }=\;{\Omega^{\prime }}_{7}\\ N=\Omega_{8},N^{\prime }=\Omega_{9},N^{\prime \prime }=\;{\Omega^{\prime }}_{9} \end{array}$$

(27) $${\Omega^{\prime }}_{3}=\frac{\begin{array}{c} \varepsilon \left(2-\beta \right)\left(\frac{\eta }{2}\Omega_{3}+\Omega_{2}-1\right)+M\left(2-\beta \right)\left(\Omega_{2}-1\right)-\lambda \left(2-\beta \right)\left(\Omega_{2}-1\right)-\\ Fr\left(2-\beta \right)\left(\Omega_{2}-1\right)-\beta \left(1-\Omega_{2}^{2}\right)-\Omega_{1}\Omega_{3} \end{array}}{\left(\left(1+W\right)-W\gamma \Omega_{3}^{2}\right)}$$

(28) $${\Omega^{\prime }}_{5}=\frac{Pr\left(2\Omega_{2}\Omega_{4}-\Omega_{1}\Omega_{5}\right)-Pr\left(Nb\Omega_{5}\Omega_{7}+Nt\Omega_{5}^{2}-\left(2-\beta \right)\frac{1}{2}\varepsilon \left(\eta \Omega_{5+3\Omega_{4}}\right)\right)}{\left(1+\frac{4}{3}Rd\right)}$$

(29) $${\Omega^{\prime }}_{7}=ScCh\Omega_{6}-Sc\left(\left(\Omega_{1}\Omega_{7}-2\Omega_{2}\Omega_{6}\right)-\frac{\varepsilon }{2}\left(2-\beta \right)\left(\eta \Omega_{7}+3\Omega_{6}\right)\right)-\frac{Nt}{Nb}{\Omega^{\prime }}_{5},\;\;$$

(30) $${\Omega^{\prime }}_{9}=Pe\Omega_{7}\Omega_{12}-Lb\left(\Omega_{1}\Omega_{9}-2\Omega_{2}\Omega_{8}-\frac{\varepsilon }{2}\left(2-\beta \right)\left(\eta \Omega_{9}+3\Omega_{8}\right)\right)+Pe\left(\Omega_{8}+\varpi \right){\Omega^{\prime }}_{7}.\;\;$$

The boundary conditions are given as

Static wedge

(31) $$\left.\begin{array}{c} \Omega_{1}\left(0\right)=S,\Omega_{2}\left(0\right)=0,\Omega_{3}\left(0\right)=-\gamma \left(1-\Omega_{4}\left(0\right)\right),\Omega_{7}\left(0\right)=1,\Omega_{8}\left(0\right)=1\\ \Omega_{2}\left(\infty \right)=1,\Omega_{4}\left(\infty \right)=0,\Omega_{6}\left(\infty \right)=0,\Omega_{8}\left(\infty \right)=0. \end{array}\right\}$$

Moving wedge

(32) $$\left.\begin{array}{c} \Omega_{1}\left(0\right)=S,\Omega_{2}\left(0\right)=\lambda ,\Omega_{3}\left(0\right)=-\gamma \left(1-\Omega_{4}\left(0\right)\right),\Omega_{7}\left(0\right)=1,\Omega_{8}\left(0\right)=1\\ \Omega_{2}\left(\infty \right)=1,\Omega_{4}\left(\infty \right)=0,\Omega_{6}\left(\infty \right)=0,\Omega_{8}\left(\infty \right)=0. \end{array}\right\}$$

The boundary condition in equation (32) is exploited through the use of a finite value$\;\eta_{max}$ as given

$$f^{\prime }\left(\eta_{max}\right)\to 0,\;\theta \left(\eta_{max}\right)\to 0,\phi \left(\eta_{max}\right)\to 0,N\left(\eta_{max}\right)\to 0.$$

The step is taken Δη=0.001 and the convergent principles are $10^{-6}$ for the required accuracy.

## 4. Result and Analysis

The consequence of several emerging parameters on velocity $f^{\prime }\left(\eta \right)$, $\theta \left(\eta \right)$, concentration$\;\phi \left(\eta \right)$, $N\left(\eta \right)\;$are studied. In this study, the flow is elaborated through $\left(i\right)\;$dwindling wedge (λ<0)$\;\left(ii\right)$ static wedge (λ=0.0) $\left(iii\right)$ stretching wedge (λ>0.0). Table 1 sees ready to verify the accuracy of our result. This table presents the comparision of $-f^{\prime \prime }\left(0\right)$ for several values of β with Khan [48]. Our results are in excellent accord. Table 2, Table 3, Table 4 and Table 5 show the impact of skin friction, the Nu_x_, the Sh_x_, and motile microorganism density.

**Figure 2:**
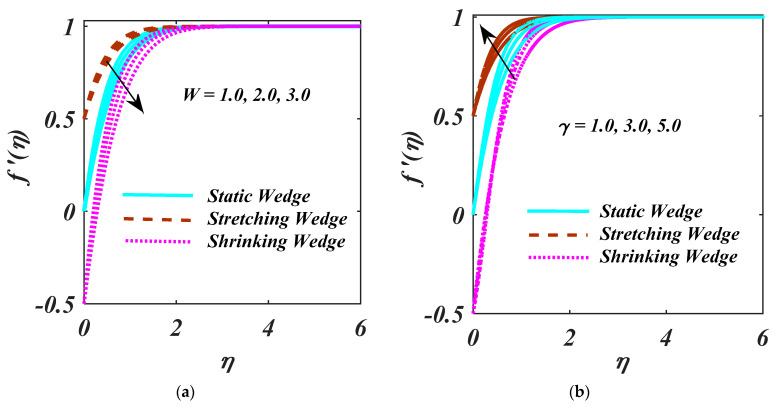
(**a**,**b**) $f^{\prime }\left(\eta \right)$ impact on W and γ.

$f^{\prime }\left(\eta \right)\;\;displays\;$the larger value of W and γ  for (λ>0), (λ=0), and (λ<0) are described in Figure 2a,b. Larger values for W reduce $f^{\prime }\left(\eta \right)$. The behavior of γ on the $f^{\prime }\left(\eta \right)$ is displayed in Figure 2b. With the lower velocity due to the larger magnitude of γ, it is clear that the $f^{\prime }\left(\eta \right)$ is larger for the case of a (λ>0) whereas compared to (λ=0), and (λ<0). The velocity ratio λ parameter’s physical relevance sees the proportion of the extending velocity to velocity of ambient. Velocity ratio parameter is increased, and the extending velocity increases faster than the ambient velocity. As interpreted in Figure 2a,b, the fluid rate is improved by the velocity ratio, which also reduces the momentum boundary layer.

**Figure 3:**
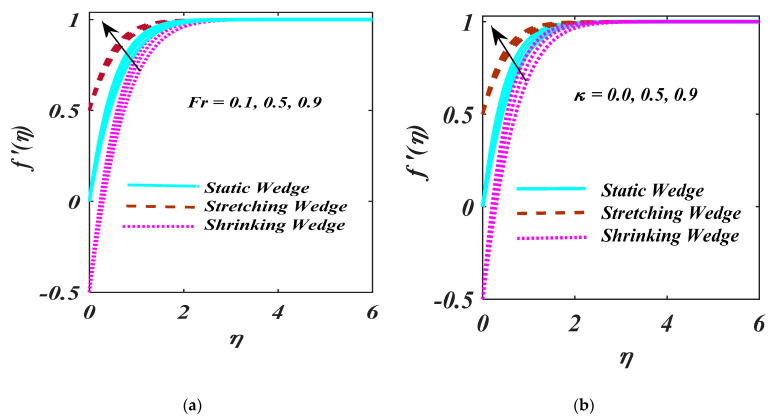
(**a**,**b**)$\;f^{\prime }\left(\eta \right)$ impacton Fr and κ.

Figure 3a,b illustrates how Fr and κ affect the velocity field. It is investigated whether the velocity field exhibits a decelerating trend as the variances Fr are increased. This is because the greater values of Fr produce resistance in a liquid flow, which reduces the velocity. The impact of the κ on the velocity distribution is elucidated in Figure 3b. The liquid’s velocity shrinks on more valuations of the κ. Due to the existence of a permeable medium, the liquid’s motion is halted, which causes a fall-off in liquid velocity.

**Figure 4:**
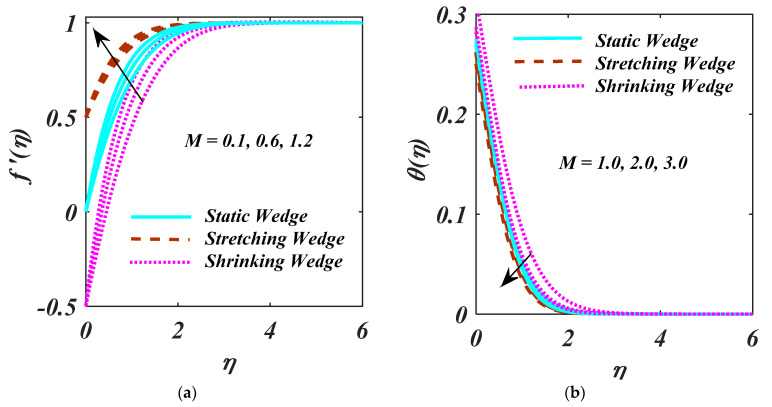

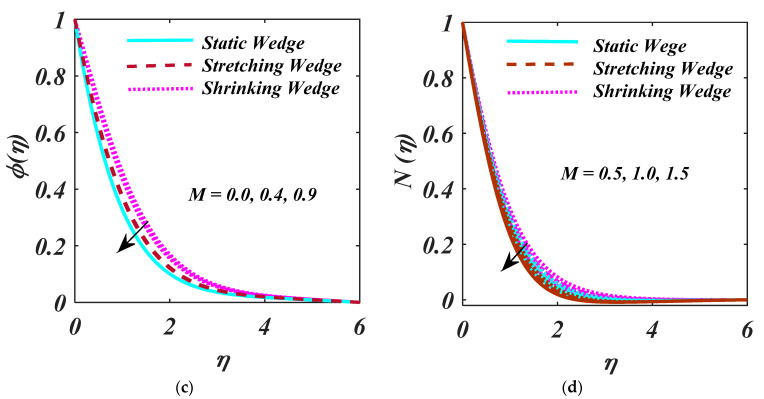
(**a**–**d**) $f^{\prime }\left(\eta \right)$, $\theta \left(\eta \right)$, $\phi \left(\eta \right)$, $N\left(\eta \right)$ impact on M.

The characteristics of M on the $f^{\prime }\left(\eta \right)$, $N\left(\eta \right)$ $\theta \left(\eta \right)$, $\phi \left(\eta \right)$ is demonstrated in Figure 4a–d. Less velocity due to the larger values of M. The increment of *M* develops a strong Lorentz force that decays the $f^{\prime }\left(\eta \right)$. Furthermore, it is clear that (λ>0) was as compared with (λ=0), and (λ<0). Augmention in *M* on $\theta \left(\eta \right)$ as seen in Figure 4b. The variation of magnetic factor on $\phi \left(\eta \right)$ and $N\left(\eta \right)$ is employed in Figure 4c,d. The leading value of M, both the $\phi \left(\eta \right)$, and $N\left(\eta \right)$. The boiling liquid moves downstream as the velocity ratio rises, bringing the fluid’s temperature down (see Figure 4b).

**Figure 5:**
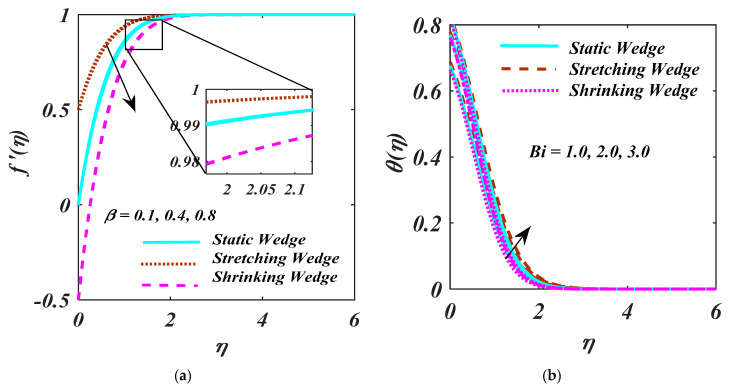
(**a**,**b**)$\;f^{\prime }\left(\eta \right)$ and $\theta \left(\eta \right)$ impact on β and Bi.

Attribute β and Bi are illustrated for (λ>0), (λ=0), and (λ<0) on thermal and velocity in Figure 5a,b. The upsurge value of the β for $f^{\prime }\left(\eta \right)$ is seen in Figure 5a. The larger values of β, the $f^{\prime }\left(\eta \right)$ has augmented. Increasing β accelerate the pressure gradient that enhances the momentum boundary layer thickness. The change of Bi over the $\theta \left(\eta \right)$ is shown in Figure 5b. A larger Bi causes $\theta \left(\eta \right)$. The larger surface as well as strong convection with a higher thermal layer $\theta \left(\eta \right)$ are explained through increasing Bi.

**Figure 6:**
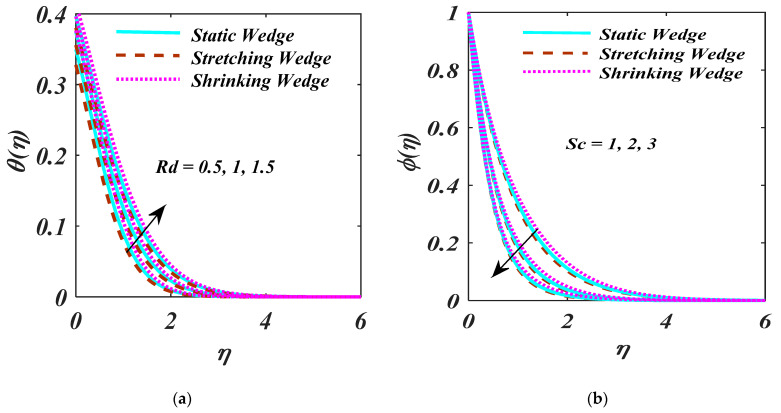
(**a**,**b**)$\;\theta \left(\eta \right)$ and $\phi \left(\eta \right)$ on Rd and Sc.

The curve of Rd  and Sc  over the $\theta \left(\eta \right)$ is portrayed in Figure 6a,b. Figure 6a portrays that the $\theta \left(\eta \right)$ produces more obvious causes for the larger values of Rd. The mean absorption coefficient reduces with the larger Rd and heat flux. Therefore, the temperature of the fluid is upsurged higher rate of radiative heat transport. Figure 6b exhibits the characteristics of the Sc  over the $\phi \left(\eta \right)$. It is clear that $\phi \left(\eta \right)$ and related thickness devalue as an upsurge Sc. Actually, Sc is explaining molecular diffusivity over a larger magnitude of the Sc.

**Figure 7:**
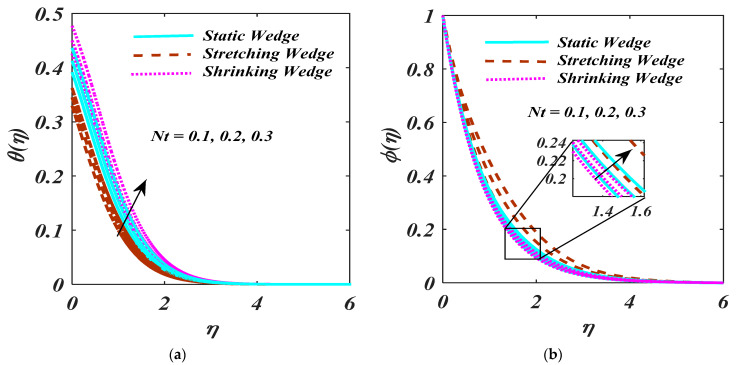
(**a**,**b**) $\theta \left(\eta \right)$ and $\phi \left(\eta \right)$ impact on Nt.

The fluctuation in Nt is exhibited in Figure 7a,b. As the $\theta \left(\eta \right)$ and $\phi \left(\eta \right)$ enhance for the rising magnitude of Nt, as the $\theta \left(\eta \right)$ and $\phi \left(\eta \right)$ inclined, causes extra particles pushed away from the warm stretchable surface to cold.

**Figure 8:**
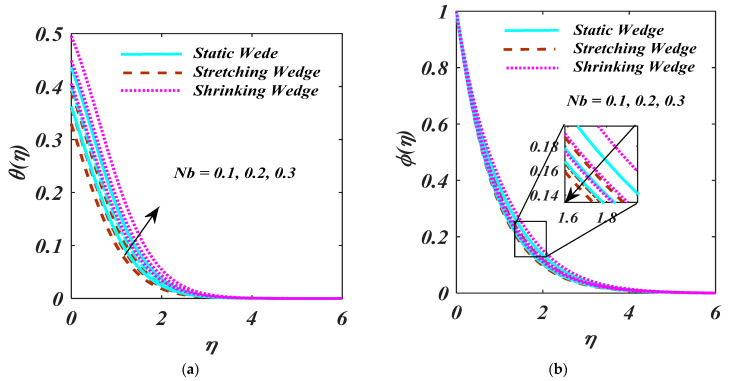
(**a**,**b**) $\theta \left(\eta \right)$ and $\phi \left(\eta \right)$ impact on Nb.

Figure 8a,b reports the high values of Nb and Nt for $\theta \left(\eta \right)$ and $\phi \left(\eta \right)$. The temperature field $\theta \left(\eta \right)\;\mathrm{is}\;$upswing due to larger Nb. Betterment of temperature profile due to the prominent between particle motion produces extra heat with the Nb upgrades. The higher magnitude of Nt causesfor the growth of nanoparticle concentration. It is interpreted that the higher the magnitude of the Nt, the extra particles are moved far from the hotter region which boosts the increment of nanoparticles concentration.

**Figure 9:**
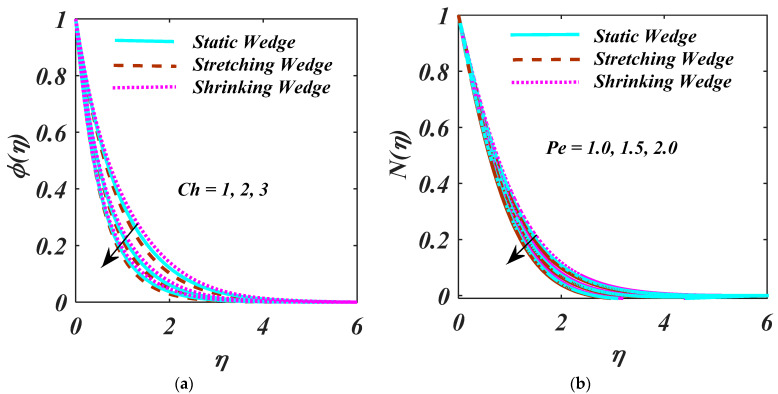
(**a**,**b**)$\;\phi \left(\eta \right)$ and $N\left(\eta \right)$ impact on Ch and Pe on.

**Figure 10:**
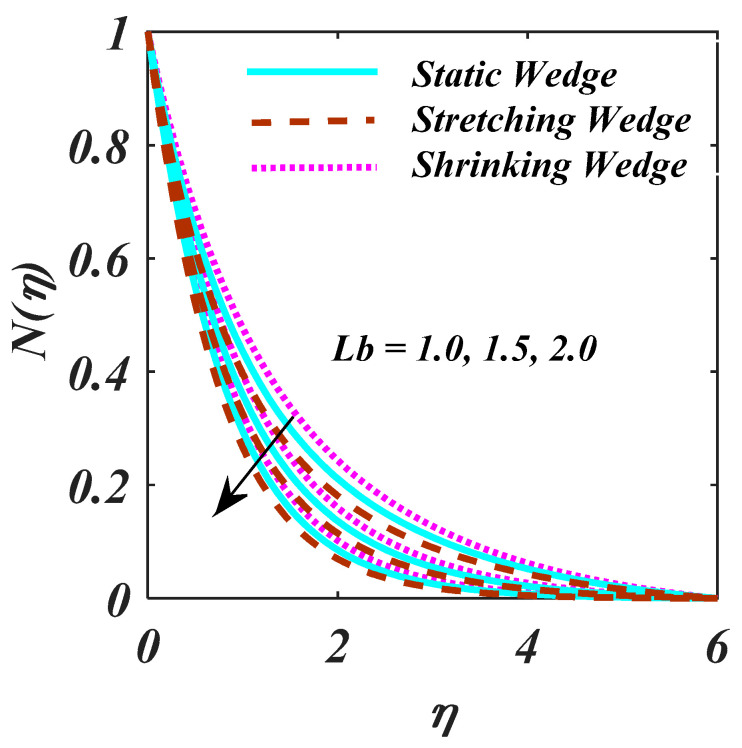
Plot of Lb on $N\left(\eta \right)$.

**Figure 11:**
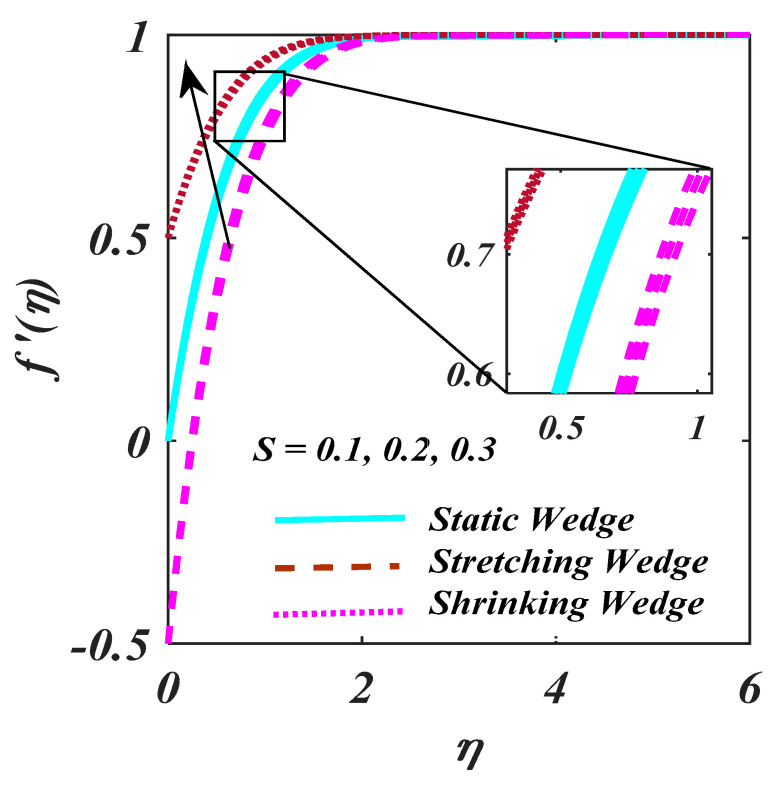
Plot of S on $f^{\prime }\left(\eta \right)$.

The response of Ch and Pe via $\phi \left(\eta \right)$ and $N\left(\eta \right)$ are examined in Figure 9a,b. Increment in Ch results reduction in $\phi \left(\eta \right)$ as designed Figure 9a,b indicates the higher magnitude of Pe  obtained to decline the $N\left(\eta \right)$. In fact, Pe comprises the reverse drifts microorganism diffusivity since the $N\left(\eta \right)$ decreases. Figure 10 investigates the higher magnitude of Lb is reports to the bigger diffusion rate. Figure 11 exposed the S over $f^{\prime }\left(\eta \right)$. S creates the fluid to abandon the system via a surface causing lessening in this way.

**Figure 12:**
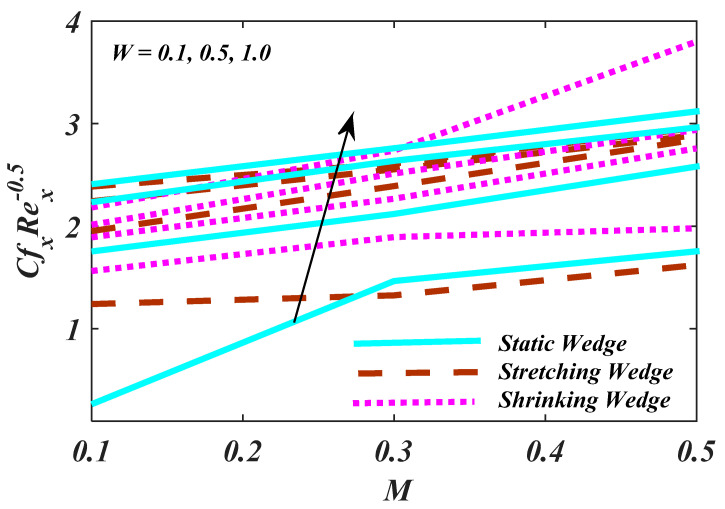
Plot of Rex1/2Cf with variations in M and W.

**Figure 13:**
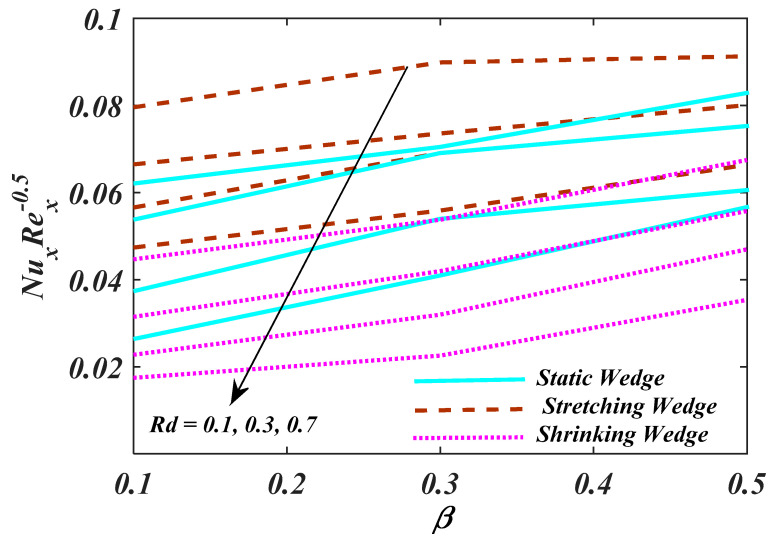
Plot of Rex−1/2Nu with variations in β and Rd.

**Figure 14:**
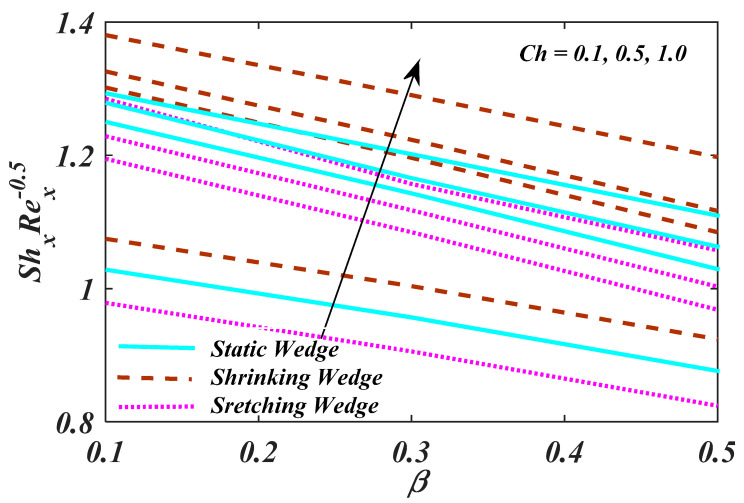
Plot of Rex−1/2Sh with variations in β and Ch.

**Figure 15:**
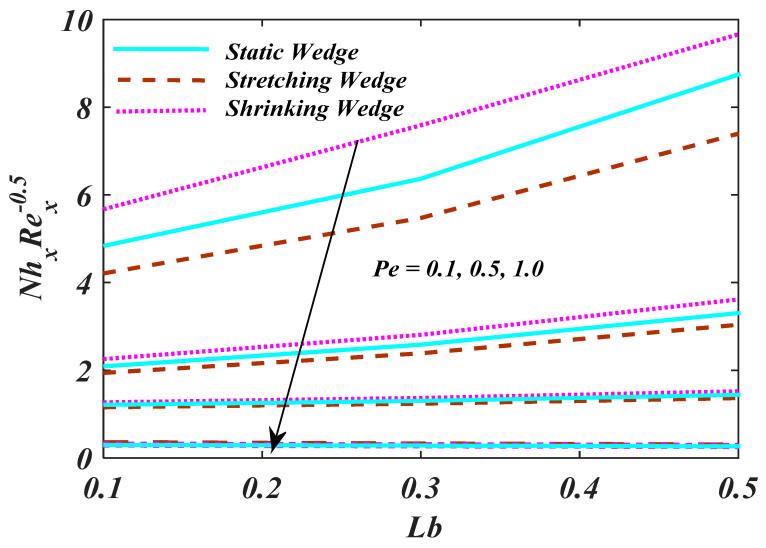
Plot of Rex−1/2Nn with variations in Pe and Lb.

**Figure 16:**
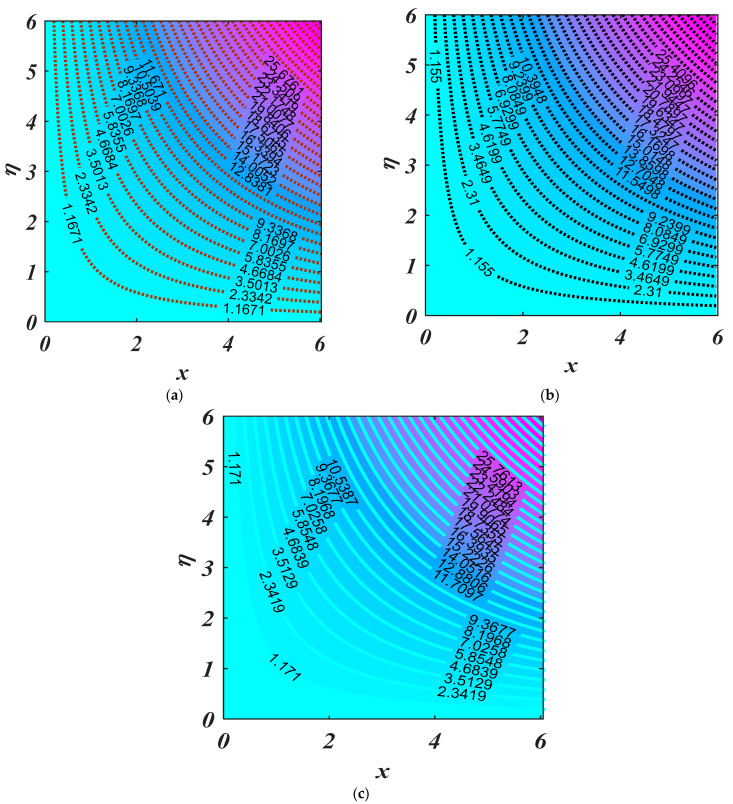
Streamline for (**a**) shrinking Wedge (−2.5); (**b**) stretching Wedge (2.5); (**c**) static Wedge (0).

Figure 12 demonstrates the characteristics of magnetic parameter M, Unsteady parameter ε, Darcy parameter Fr, Porosity parameter κ and fluid parameter W on the $Cf_{x}Re_{x}^{1/2}$, The $Cf_{x}Re_{x}^{1/2}$ is an increasing function of W. The influence of Rd and β  on $NuRe_{x}^{-1/2}$ is explained in Figure 13. Heat transport decline with larger values of M and β. Figure 14 offers the effects of Ch, Sc, Nt, Nb, β and ε for Rex−1/2Sh. Ch and β have a positive influence for Rex−1/2Sh. The (Rex−1/2Nh) on the surface is shown in Figure 15 which shows the effects of Pe, Lb, ε and ϖ. This graph depicts that the motile density drops with growing values of Pe. Figure 16a–c depicts a fascinating enactment of the streamline in the actuality of λ=0, λ<0 and λ>0. The arrangements portray that the streamlining is more manifest and split into three segments. The pattern is very simple and follows the flow field.

## 5. Final Remarks

This study includes the mechanism of bioconvection for Eyring–Powell nanofluid as of magnetic field with three different angles due to Darcy–Forccheimer. Temperature and concentration constitutive equations are used to explore the Buongiorno model of the nanofluid. The Bvp4c technique is considered to handle ordinary differential equations (ODEs). The boundary value problem bvp4c is a numerical code for a computationally intensive solution; it is used here to track the solution of the developed model. The motive of this work is to reduce drag friction and strengthen the rate of heat and mass transfer. Recently, Abbasi [49] published research on blood flow with differently shaped nanoparticles in micromachines. The main points are listed as

- Larger values of W and γ shrink the velocity profile.
- A larger Forchheimer number Fr depicts the decreasing behaviour for the velocity profile.
- Rising values of *M* enhance the stretching wedge of velocity.
- An augmentation of Nb leads to a reduction in the liquid concentration;
- Larger values of the Biot number Bi show an increasing behaviour for temperature, but the opposite trend is noticed for the Ch.
- By increasing the magnitude of the Pe and Lb, there is reduction behaviour.
- The density of the $N\left(\eta \right)$ and the$\;\phi \left(\eta \right)$ as the fluid parameters elevated, while the rate of the skin friction upsurges.

The major application of the current study can be found in aerospace engineering.

## Figures and Tables

**Table 1:** An assessment value of $-f^{\prime \prime }\left(0\right)$ for dissimilar values of β with reference [48] when W=0, γ=0, M=S=0.

β	Khan et al. [48]	Current Outcomes	% Error
0.0	0.4696005	0.4695999	$6\times 10^{-5}$
0.1	0.5870353	0.5870352	$1\times 10^{-5}$
0.3	0.7747546	0.7747545	$1\times 10^{-5}$
0.5	0.9276800	0.9276799	$1\times 10^{-5}$
1.0	1.2325880	1.2325876	$4\times 10^{-5}$

**Table 2:** Skin friction for various physical variables.

W	β	ε	γ	Fr	κ	M	CfRex1/2 λ=0	CfRex1/2 λ=−2.5	CfRex1/2 λ=2.5
0.1	0.1	0.1	0.3	0.1	0.1	0.1	0.2637	1.5630	1.2408
0.5							1.4654	1.8936	1.3256
1.0							1.7553	1.9796	1.6211
0.1	0.2	0.4	0.3	0.3	0.2	0.3	1.7534	1.8921	1.9061
0.5							2.1194	2.2681	2.3521
1.0							2.5818	2.7586	2.8408
0.1	0.3	0.7	0.3	0.6	0.3	0.6	2.2342	2.0140	2.2460
0.5							2.6377	2.5138	2.5436
1.0							2.9625	2.9398	2.8549
0.1	0.4	0.9	0.3	0.9	0.4	0.9	2.4091	2.1823	2.3869
0.5							2.7604	2.7382	2.6117
1.0							3.1190	3.7998	2.9589

**Table 3:** Nusselt number for various physical variables.

Rd	ε	Nt	Nb	Pr	β	NuxRex1/2 ***λ* = 1**	NuxRex1/2 ***λ* = − 2.5**	NuxRex1/2 ***λ* = 2.5**
0.1	0.1	0.3	0.6	1.0	0.4	0.0447	0.0621	0.0796
0.3						0.0538	0.0705	0.0899
0.7						0.0675	0.0829	0.0913
0.1	0.2	0.5	0.8	2.0	0.6	0.0358	0.0538	0.0665
0.3						0.0420	0.0691	0.0736
0.7						0.0558	0.0753	0.0801
0.1	0.3	0.7	1.0	3.0	0.8	0.0228	0.0374	0.0566
0.3						0.0320	0.0540	0.0691
0.7						0.0470	0.0606	0.0753
0.1	0.4	0.9	1.2	4.0	1.0	0.0176	0.0264	0.0474
0.3						0.0226	0.0410	0.0559
0.7						0.0354	0.0567	0.0664

**Table 4:** Sherwood number for various physical variables.

Ch	ε	Sc	Nt	Nb	β	ShxRex1/2 λ=0	ShxRex1/2 λ=−2.5	ShxRex1/2 λ=2.5
0.1	0.1	0.5	0.4	0.2	0.4	1.0284	0.9785	1.0747
0.5						0.9567	0.9057	1.0037
1.0						0.8763	0.8241	0.9241
0.1	0.3	1.0	0.6	0.4	0.6	1.2502	1.1948	1.3017
0.5						1.1428	1.0846	1.1963
1.0						1.0292	0.9682	1.0846
0.1	0.4	1.5	0.8	0.6	0.8	1.2788	1.2286	1.3259
0.5						1.1658	1.1175	1.2233
1.0						1.0630	1.0029	1.1167
0.1	0.6	2.0	1.0	0.8	1.0	1.2932	1.1849	1.3304
0.5						1.2017	1.1571	1.1603
1.0						1.1095	1.0573	1.1557

**Table 5:** Motile density microorganisms for various physical variables.

Pe	Lb	ϖ	∈	β	NhxRex1/2 λ=0	NhxRex1/2 λ=−2.5	NhxRex1/2 λ=2.5
0.1	0.5	0.2	0.1	0.4	0.2970	0.3054	0.3381
0.5					0.2804	0.2886	0.3073
1.0					0.2653	0.2734	0.2734
0.1	1.0	0.6	0.3	0.6	1.2121	1.2694	1.1572
0.5					1.3011	1.3679	1.2378
1.0					1.4400	1.5206	1.3649
0.1	1.5	1.0	0.6	0.8	2.0884	2.2555	1.9393
0.5					2.5836	2.8085	2.3871
1.0					3..3051	3.6163	3.0382
0.1	2.0	1.4	0.9	1.0	4.8372	5.6726	4.2083
0.5					6.3681	7.5882	5.4749
1.0					8.7454	9.6614	7.3976

## Data Availability

All the data are clearly available in the manuscript.

## Nomenclature

W	fluid variable
γ	fluid variable
*M*	Magnetic variable
∈	Unsteady variable
λ	velocity variable with ration
Fr	Darcy Parameter
*K*	Porosity variable
*Pr*	Prandtl number
*Nt*	Thermophoresis variable
*Nb*	Brownian motion
β	Pressure gradient parameter
*Bi*	Biot number
*Rd*	Radiation parameter
*Ch*	Chemical reaction parameter
*Sc*	Schmidt number
*Pe*	Peclet number
Lb	Bioconvection Lewis number
$\theta_{w}$	Temperature ratio parameter
$C_{f}$	Skin fiction coefficient
$Nu_{x}$	Heat transport coefficient (Nusselt)
$Sh_{x}$	Mass Nusselt number
$Nh_{x}$	motile density
η	Similarity parameter
g	Acceleration due to Gravity
u,v	Velocity
τ	heat capacity with effectivness
υ	viscosity of Kinematic
S	Stretching/shrinking variable
$\alpha_{m}$	Thermal diffusivity
$D_{B}$	Mass diffusivity
$D_{T}$	Thermophoresis diffusivity
${\left(\rho d\right)}_{f}$	heat capacity of fluid
${\left(\rho d\right)}_{p}$	Nanoparticles heat capacity
$B_{o}$	Magnetic field strength
$T,T_{\infty }$	Temperature of fluid
$C,C_{\infty }$	Concentration susceptibility
$T_{w}$	Variable temperature
$C_{w}$	Variable concentration
ρ	density of fluid
$U_{e}$	Free stream velocity
